# Assessment of the Performance Consistency of an Adverse Outcome Prediction Tool for Patients Hospitalized With COVID-19

**DOI:** 10.1001/jamanetworkopen.2021.18413

**Published:** 2021-07-27

**Authors:** Victor M. Castro, Thomas H. McCoy, Roy H. Perlis

**Affiliations:** 1 Center for Quantitative Health, Division of Clinical Research, Massachusetts General Hospital, Boston; 2 Research Information Science and Computing, Mass General Brigham, Somerville, Massachusetts

## Abstract

This prognostic study reports on the performance of a previously validated COVID-19 severity prediction tool when applied to data during the second wave of the pandemic.

## Introduction

The challenge of managing limited resources during the COVID-19 pandemic has sparked efforts to stratify risk among hospitalized patients.^1^ Few risk models have been validated or investigated for potential bias^2^ even though inpatient populations, treatments, and outcomes for COVID-19 have changed over time. We previously^3^ reported and validated a risk prediction tool based on COVID-19 hospitalizations during the initial wave of the pandemic. In this study, we report the performance of that same model on subsequent data from 6 hospitals collected during the second wave of patients with COVID-19.

## Methods

In this prognostic study, we included individuals aged 18 years or older who were hospitalized at 1 of 2 academic medical centers and 4 community hospitals from June 7, 2020, through January 22, 2021, with a positive polymerase chain reaction test for SARS-CoV-2 within 5 days of admission, excluding those with an outcome on the day of hospitalization. The study protocol was approved by the Mass General Brigham Human Research Committee, which waived informed consent given that this is a minimal risk study using deidentified data. The Transparent Reporting of a Multivariable Prediction Model for Individual Prognosis or Diagnosis (TRIPOD) reporting guideline for validation studies was applied.

Features of hospital course were extracted from the Mass General Brigham Data Registry^4^ and the Enterprise Data Warehouse, including laboratory values and high and low flags. The Charlson Comorbidity Index was calculated using coded *International Statistical Classification of Diseases and Related Health Problems, Tenth Revision *(*ICD-10*) diagnostic codes.^5^ Race and ethnicity were defined by patient self-report using US Census categories and were included to allow assessment of bias in model performance.

Patients were followed up from admission to hospital discharge or death, with follow-up censored at discharge. Primary outcomes were (1) a composite severe illness outcome, including admission to the intensive care unit (ICU), mechanical ventilation, or mortality and (2) mortality. Coefficients from our previously reported least absolute shrinkage and selection operator risk models were applied to estimate the probability of each outcome without recalibration; these coefficients were drawn from sociodemographic features, the comorbidity index, and laboratory values.^3^ We applied median imputation of missing data. We characterized model performance with standard metrics of discrimination and calibration. All analyses were conducted with R version 4 (R Project for Statistical Computing).

## Results

Features of the new cohort are summarized in Table 1 and compared with those of the previously reported cohort in which the predictive model was trained. For the 2892 individuals in the new cohort, the mean (SD) age was 63.0 (19.1) years; they included 1460 (50.5%) women, 673 (23.3%) Hispanic individuals, and 344 (11.9%) Black individuals. The mean (SD) length of hospital stay was 6.2 (5.3) days; 126 patients (4.4%) required an ICU stay and 68 (2.4%) mechanical ventilation, while 167 (5.8%) died prior to discharge. Overall model performance for mortality included an area under the receiver operating characteristic curve (AUC) of 0.83 (95% CI, 0.80-0.87), with a positive predictive value (PPV) of 0.22 and a negative predictive value (NPV) of 0.98 when using a cutoff corresponding to the highest 20% of predicted risk derived in the training set. By comparison, in the original model period,^3^ AUC was 0.85; PPV, 0.46; and NPV, 0.97. For the composite severe outcome, AUC was 0.78 (95% CI 0.75-0.81); PPV, 0.25; and NPV, 0.95 in the top 20% risk group vs an AUC of 0.81, PPV of 0.55, and NPV of 0.91 in the original period.^3^ Among subgroups (Table 2), model discrimination for both outcomes was generally similar among sex and race/ethnicity groups but poorer for younger age groups.

**Table 1.** Sociodemographic and Illness Severity Comparison Between the Initial Model Training COVID-19 Admission Cohort and the Subsequent Admissions Used to Evaluate the Model^a^

Characteristic	Patients, No. (%)		*P* value
	Initial training, Mar 11 to Jun 6, 2020 (n = 1877)	Replication, Jun 7, 2020, to Jan 22, 2021 (n = 2892)	
Community hospital admission	885 (47.1)	1464 (50.6)	.02
Age, y			
Mean (SD)	62.0 (19.3)	63.01 (19.1)	.08
Range	18-102	18-102	
Median (IQR)	63 (48-78)	65 (50-78)	
Age group, y			
<50	514 (27.4)	698 (24.1)	.02
50-69	625 (33.3)	954 (33.0)	
≥70	738 (39.3)	1240 (42.9)	
Gender			
Male	983 (52.4)	1432 (49.5)	.05
Female	894 (47.6)	1460 (50.5)	
Race			
Asian	70 (3.7)	118 (4.1)	<.001
Black	209 (11.1)	344 (11.9)	
Other^b^	493 (26.3)	588 (20.3)	
White	1105 (58.9)	1842 (63.7)	
Hispanic ethnicity	563 (30.0)	673 (23.3)	<.001
Charlson Comorbidity Index			
Mean (SD)	2.6 (3.254)	2.9 (3.5)	<.001
Range	0-21	(0-21	
Median (IQR)	1 (0-4)	2 (0-5)	
Hospital length of stay, d			
Mean (SD)	7.5 (7.6)	6.2 (5.3)	<.001
Range	1-66	1-55	
Median (IQR)	5 (3-9)	5 (3-7)	
ICU admission	161 (8.6)	126 (4.4)	<.001
Mechanical ventilation	129 (6.9)	68 (2.4)	<.001
Discharged to SNF or rehabilitation facility	798 (42.5)	654 (22.6)	<.001
Death	209 (11.1)	167 (5.8)	<.001
Severe COVID-19 outcome^c^	338 (18.0)	241 (8.3)	<.001

Abbreviations: ICU, intensive care unit; IQR, interquartile range; SNF, skilled nursing facility.

^a^ The training data set consisted of the initial surge of COVID-19 cases in eastern Massachusetts, whereas the replication cohort included the summer nadir and second wave in the fall of 2020.

^b^ The other race category included patients who self-reported multiracial or other race and patients whose race is unknown.

^c^ Severe COVID-19 outcome refers to the composite severe illness outcome, including admission to the ICU, mechanical ventilation, or mortality.

**Table 2.** Discrimination and Calibration Metrics of the COVID-19 Severity and Mortality Prediction Model by Subgroup

Subgroup	Original testing cohort, March to June 2020							Evaluation cohort, June 2020 to January 2021						
	Patients, No.	Patients with severe COVID-19	AUC (95% CI)	Specificity^a^	Sensitivity^a^	PPV^a^	NPV^a^	Patients, No.	Patients with severe COVID-19	AUC (95% CI)	Specificity^a^	Sensitivity^a^	PPV^a^	NPV^a^
**COVID-19 severity prediction model**														
Academic medical center	348	54	0.83 (0.77-0.89)	0.88	0.56	0.47	0.92	1428	106	0.75 (0.70-0.80)	0.86	0.54	0.23	0.96
Community hospital	275	60	0.79 (0.73-0.86)	0.86	0.55	0.52	0.87	1464	135	0.80 (0.77-0.84)	0.83	0.58	0.26	0.95
Female	333	52	0.77 (0.71-0.84)	0.89	0.46	0.43	0.90	1460	91	0.77 (0.72-0.82)	0.89	0.48	0.23	0.96
Male	290	62	0.85 (0.79-0.90)	0.86	0.63	0.55	0.89	1432	150	0.78 (0.74-0.81)	0.80	0.61	0.26	0.95
Age, y														
<50	118	3	0.70 (0.52-0.88)	0.98	0.00	0.00	0.97	698	17	0.64 (0.48-0.79)	0.98	0.18	0.20	0.98
50-69	258	40	0.83 (0.77-0.89)	0.89	0.42	0.40	0.89	954	58	0.69 (0.62-0.76)	0.86	0.38	0.15	0.96
≥70	247	71	0.76 (0.69-0.83)	0.79	0.65	0.55	0.85	1240	166	0.77 (0.73-0.80)	0.74	0.66	0.29	0.93
Asian	25	4	0.88 (0.71-1.00)	0.81	0.75	0.43	0.94	118	10	0.86 (0.75-0.98)	0.90	0.50	0.31	0.95
Black	215	37	0.80 (0.72-0.89)	0.87	0.57	0.48	0.91	344	20	0.74 (0.63-0.85)	0.84	0.55	0.17	0.97
Other^b^	139	26	0.80 (0.72-0.88)	0.83	0.54	0.42	0.89	588	30	0.77 (0.68-0.86)	0.89	0.43	0.18	0.97
White	244	47	0.83 (0.76-0.89)	0.91	0.53	0.58	0.89	1842	181	0.78 (0.74-0.81)	0.83	0.59	0.27	0.95
Hispanic	115	20	0.78 (0.68-0.88)	0.86	0.50	0.43	0.89	673	30	0.74 (0.63-0.84)	0.90	0.33	0.14	0.97
Not Hispanic	508	94	0.82 (0.77-0.87)	0.88	0.56	0.51	0.90	2219	211	0.78 (0.75-0.81)	0.83	0.59	0.27	0.95
**Subgroup**	**Original testing cohort (March-June 2020)**							**Evaluation cohort (June 2020-January 2021)**						
	**Patients, No.**	**Died in hospital**	**AUC (95% CI)**	**Specificity**^a^	**Sensitivity**^a^	**PPV**^a^	**NPV**^a^	**No.**	**Died in hospital**	**AUC (95% CI)**	**Specificity**^a^	**Sensitivity**^a^	**PPV**^a^	**NPV**^a^
**COVID-19 mortality prediction model**														
Academic medical center	348	33	0.87 (0.82-0.92)	0.85	0.61	0.30	0.95	1428	70	0.80 (0.74-0.86)	0.87	0.60	0.19	0.98
Community hospital	275	50	0.82 (0.76-0.88)	0.84	0.64	0.48	0.91	1464	97	0.86 (0.82-0.89)	0.84	0.71	0.24	0.98
Female	333	37	0.83 (0.77-0.89)	0.85	0.54	0.32	0.94	1460	60	0.82 (0.76-0.88)	0.88	0.57	0.17	0.98
Male	290	46	0.86 (0.81-0.91)	0.84	0.70	0.45	0.94	1432	107	0.83 (0.79-0.87)	0.83	0.72	0.25	0.97
Age, y														
<50	118	0	NA	NA	NA	NA	NA	698	7	0.56 (0.31-0.80)	0.99	0.00	0.00	0.99
50-69	258	23	0.83 (0.76-0.90)	0.91	0.35	0.28	0.93	954	27	0.70 (0.60-0.80)	0.93	0.22	0.09	0.98
≥70	247	60	0.76 (0.69-0.83)	0.68	0.73	0.42	0.89	1240	133	0.81 (0.78-0.85)	0.70	0.79	0.24	0.97
Asian	25	3	0.92 (0.76-1.00)	0.86	0.67	0.40	0.95	118	6	0.90 (0.80-0.99)	0.91	0.50	0.23	0.97
Black	215	28	0.87 (0.80-0.93)	0.84	0.71	0.41	0.95	344	11	0.90 (0.84-0.96)	0.88	0.73	0.16	0.99
Other^b^	139	17	0.83 (0.74-0.91)	0.84	0.53	0.31	0.93	588	17	0.84 (0.73-0.95)	0.92	0.53	0.17	0.99
White	244	35	0.84 (0.77-0.90)	0.86	0.60	0.41	0.93	1842	133	0.81 (0.77-0.85)	0.82	0.68	0.23	0.97
Hispanic	115	14	0.84 (0.75-0.92)	0.89	0.43	0.35	0.92	673	16	0.75 (0.60-0.91)	0.93	0.44	0.13	0.99
Not Hispanic	508	69	0.85 (0.81-0.89)	0.84	0.67	0.39	0.94	2219	151	0.83 (0.80-0.87)	0.83	0.69	0.23	0.97

Abbreviations: NA, not applicable; NPV, negative predictive value; PPV, positive predictive value; AUC, area under the receiver operating characteristic curve.

^a^ Specificity, sensitivity, PPV, and NPV are reported for the top 20% of risk score defined in the original training set.

^b^ The other race category included patients who self-reported multiracial or other race and patients whose race is unknown.

## Discussion

Applying a previously validated model to 2892 new COVID-19 admissions in the same 6 hospitals, we found that model performance decreased only modestly from the initial validation study.^3^ A key exception was PPV, likely reflecting substantial diminution in mortality and mechanical ventilation between the original and the subsequent study periods. Discrimination was generally consistent across subgroups, with the notable exception of younger age groups in whom performance was poorer.

Our results indicate that the population of individuals hospitalized for COVID-19 has shifted and the prevalence of the studied outcomes changed. However, they suggest that prediction models derived earlier in the pandemic may maintain discrimination after recalibration. A limitation is the reliance on 2 health systems in the same region. Our results also illustrate the importance of investigating risk stratification models across patient subgroups as a step toward ensuring that particular groups are not adversely affected by the application of such tools, particularly in settings of potential resource constraints.

## References

[1] Knight SR, Ho A, Pius R, ; ISARIC4C investigators. Risk stratification of patients admitted to hospital with COVID-19 using the ISARIC WHO Clinical Characterisation Protocol: development and validation of the 4C Mortality Score. BMJ. 2020;370:m3339. doi:10.1136/bmj.m3339 32907855 PMC7116472

[2] Griffith GJ, Morris TT, Tudball MJ, . Collider bias undermines our understanding of COVID-19 disease risk and severity. Nat Commun. 2020;11(1):5749. doi:10.1038/s41467-020-19478-2 33184277 PMC7665028

[3] Castro VM, McCoy TH, Perlis RH. Laboratory findings associated with severe illness and mortality among hospitalized individuals with coronavirus disease 2019 in eastern Massachusetts. JAMA Netw Open. 2020;3(10):e2023934. doi:10.1001/jamanetworkopen.2020.23934 33125498 PMC7599467

[4] Nalichowski R, Keogh D, Chueh HC, Murphy SN. Calculating the benefits of a research patient data repository. AMIA Annu Symp Proc. 2006;2006:1044. 17238663 PMC1839563

[5] Charlson M, Szatrowski TP, Peterson J, Gold J. Validation of a combined comorbidity index. J Clin Epidemiol. 1994;47(11):1245-1251. doi:10.1016/0895-4356(94)90129-5 7722560

